# Association of body mass index with in-hospital major adverse outcomes in acute type A aortic dissection patients in Fujian Province, China: a retrospective study

**DOI:** 10.1186/s13019-021-01432-y

**Published:** 2021-03-23

**Authors:** Lingyu Lin, Yanjuan Lin, Qiong Chen, Yanchun Peng, Sailan Li, Liangwan Chen, Xizhen Huang

**Affiliations:** 1 Department of Nursing, Fujian Medical University, Fuzhou, China; 2 Department of Nursing, Union Hospital, Fujian Medical University, No.29 Xinquan Road, Fuzhou, 350001 Fujian Province China; 3 Department of Cardiac Surgery, Union Hospital, Fujian Medical University, Fuzhou, 350001 Fujian China

**Keywords:** Acute aortic dissection, Body mass index, Adverse outcomes

## Abstract

**Background:**

Abnormal body mass index (BMI) has been related to a higher risk of adverse outcomes in patients undergoing cardiac surgery. However, the effects of BMI in patients with acute type A aortic dissection (AAAD) on postoperative outcomes remain unclear. This study aimed to explore the relationships between BMI and in-hospital major adverse outcomes (MAO) in AAAD patients.

**Methods:**

Patients who underwent AAAD surgery at Cardiac Medical Center of Fujian Province from June 2013 to March 2020 were retrospectively evaluated. They were divided into three groups on the basis of Chinese BMI classification established by the World Health Organization: normal weight group (BMI 18.5–23.9 kg/m^2^), overweight group (BMI 24–27.9 kg/m^2^), and obese group (BMI >28 kg/m^2^). Patients’ baseline characteristics, preoperative, operative, and postoperative data were collected. A multivariable logistic regression analysis model was performed to identify the association between BMI and MAO in AAAD patients.

**Results:**

Of 777 cases, 31.9% were normal weight, 52.5% were overweight, and 15.6% were obese. A total of 160(20.6%) patients died in-hospital. There was a significant difference between the three groups for MAO (62.9% vs 72.1% vs 77.7%, respectively, *P* = 0.006). The incidence of postoperative complications did not differ among the three groups, except for postoperative bleeding, and prolonged mechanical ventilation, the proportion of which were higher in the overweight and obese groups. Besides, multivariable logistic regression analysis demonstrated that a higher risk of MAO in the overweight [odds ratios (ORs):1.475, 95%CI:1.006–2.162], and obese patients (ORs:2.147, 95%CI:1.219–3.782) with reference to the normal weight patients, and age, white blood cell, prior stroke and cardiopulmonary bypass time were also associated with in-hospital MAO (*P*<0.05).

**Conclusions:**

BMI is independently associated with higher in-hospital MAO in patients who underwent AAAD surgery.

## Background

Obesity is generally considered to be a strong risk factor for morbidity and mortality during the perioperative period in patients with heart disease [1]. Studies have reported obesity patients who underwent cardiac surgery were associated with adverse outcomes in recent years [2, 3]. However, some reports suggest that obesity is unable to increase the incidence rate of in-hospital mortality and even reduce it [4, 5]. The improved survival and functional outcomes in overweight and obese cardiac surgery patients are termed as the ‘obesity paradox’ in the literature [6].

So far, the ‘obesity paradox’ has been demonstrated in patients undergoing coronary artery bypass grafting or valve surgery [7–9]. However, it has not been fully studied in patients with acute type A aortic dissection (AAAD). Only two studies have evaluated the effect of body mass index (BMI) on postoperative clinical outcomes in patients with AAAD [10, 11], but the study was in western countries whose cases were categorized by the World Health Organization (WHO) standard. Considering ethnic differences, the WHO standard of BMI is not suitable for the Chinese population which the results of the mentioned studies might therefore not be valid for. No existing studies have reported the associations between BMI and adverse outcomes in AAAD patients undergoing surgery. Moreover, it is unknown whether the ‘obesity paradox’ in Chinese AAAD patients exists. This study adopted the Chinese BMI classification standard to determine the relationship between BMI and in-hospital major adverse outcomes (MAO) in patients with AAAD.

## Methods

### Study population

This retrospective study enrolled 777 consecutive patients aged 18–80 years at Cardiac Medical Center of Fujian Province that underwent AAAD surgery between June 2013 and March 2020.

Patients with the following conditions were excluded: (1) Pregnant women; (2) Patients with malignancy; (3) Traumatic dissection; (4) Patients lost to follow-up within 30 days; (5) Patients with incomplete clinical data.

All patients were divided into three groups on the basis of Chinese BMI classification established by WHO: BMI = 18.5–23.9 kg/m^2^(normal weight group); BMI = 24–27.9 kg/m^2^(overweight group); BMI>28 kg/m^2^(obese group). Underweight patients (BMI<18.5 kg/m^2^) were excluded to avoid biased results because of the small sample size. Ethics approval has been obtained from the ethics committee of Fujian Medical University Union Hospital (approval number:2013002) and conformed to the Declaration of Helsinki.

### Data collection

All data were collected by two investigators who had been uniformly trained, including baseline characteristics, preoperative characteristics, intraoperative, and postoperative data. The primary outcomes were frequency of in-hospital MAO and mortality. According to the consensus statement from the International Aortic Arch Surgery Study Group [12], MAO in this study were defined as the events including death, stroke, myocardial infarction, low cardiac output syndrome, arrhythmia, aortic dissection rupture, respiratory insufficiency, acute renal failure, gastrointestinal bleeding, deep sternal wound infection, septicemia, postoperative bleeding, prolonged mechanical ventilation (MV). Besides, the duration of intensive care unit (ICU) stays and hospital stays were also recorded.

### Criteria

In-hospital mortality was defined as both all-cause deaths occurring during the hospitalization or in the first 30 postoperative days despite discharge status [13]. Stroke was defined as acute onset focal neurologic deficit caused by a disturbance in blood supply to the brain that last ≥24 h [14]. Acute renal failure was defined as a creatinine level that was 3 times higher than preoperative creatinine or a new requirement for dialysis [15]. Respiratory insufficiency was defined as one of the following conditions: atelectasis, pneumonia, pulmonary edema, or acute respiratory distress syndrome, requiring intubation >72 h or a tracheostomy [12]. Prolonged mechanical ventilation was defined as the duration of mechanical ventilation (>48 h) [16].

### Statistical analysis

Continuous variables were presented as mean ± standard deviation (SD) or median (interquartile range), based on Gaussian distribution, and categorical variables were presented as numbers/percentages. Continuous variables were analyzed using analysis of variance (ANOVA) or the Kruskal-Wallis tests, and categorical variables were done using Chi-squared test or Fisher’s exact test. Multivariable logistic regression analyses were done to assess the relationship between BMI and in-hospital MAO, introducing variables with *P*<0.1 on univariate analysis and based on professional knowledge. Data were performed using the Statistical Package for the Social Science (IBM-SPSS version 23.0). *P*<0.05 was considered to be statistically significant.

## Results

**Fig. 1:**
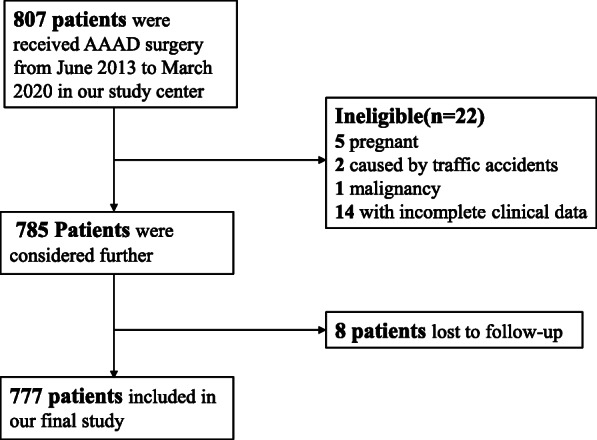
Patients’ flow chart of the cohort. *AAAD*, acute type A aortic dissection

From June 2013 to March 2020, a total of 807 AAAD patients aged 18–80 years received surgical treatment. We excluded 5 pregnant women, 2 patients caused by traffic accidents, 1 patient had malignancy, 14 with incomplete data, and 8 lost to follow-up. Finally, a cohort of 777 AAAD patients was enrolled. The flow chart of patient inclusion is demonstrated in Fig. 1.

Table 1 shows the baseline characteristics of the three groups. Among the 777 AAAD patients, 248(31.9%) were classified into the normal weight group with a BMI of (21.7 ± 1.5) kg/m^2^, 408(52.5%) were classified into the overweight group with a BMI of (25.4 ± 1.0) kg/m^2^, and 121(15.6%) patients were classified into the obese group with a BMI of (31.6 ± 6.5) kg/m^2^. The youngest age was seen in the obese group (age of 48.5 ± 11.5 years, *P*<0.001) as compared with the overweight and normal weight groups, and with a high proportion of male, drinker, and Marfan syndrome (*P*<0.05). There was no significant difference in smoker, the history of hypertension, type 2 diabetes, previous cardiac surgery, coronary artery heart disease, prior stroke, prior shock, prior resuscitation, extension of AAAD, and time from symptom onset to surgery among the three groups (*P*>0.05).

**Table 1:** Baseline Characteristics at Presentation of Patients With AAAD

Variables	normal weight group 18.5–23.9 kg/m^2^ (*N* = 248)	overweight group 24–27.9 kg/m^2^ (*N* = 408)	obese group ≥28 kg/m^2^ (*N* = 121)	*P-*value
Age, years, mean (SD)	55.7 ± 11.8	51.9 ± 10.9	48.5 ± 11.5	**< 0.001**^a^
Male, n (%)	172 (69.4)	329 (80.6)	103 (85.1)	**< 0.001**^c^
BMI (kg/m^2^), mean (SD)	21.7 ± 1.5	25.4 ± 1.0	31.6 ± 6.5	**< 0.001**^a^
History of				
Smoker, n (%)	108 (43.5)	199 (48.8)	64 (52.9)	0.201^c^
Drinker, n (%)	74 (29.8)	147 (36.0)	52 (43.0)	**0.040**^c^
Hypertension, n (%)				0.205^c^
No	84 (33.9)	114 (27.9)	27 (22.3)	
Level 1	26 (10.5)	51 (12.5)	14 (11.6)	
Level 2	44 (17.7)	66 (16.2)	18 (14.9)	
Level 3	94 (37.9)	177 (43.4)	62 (51.2)	
Type 2 diabetes, n (%)	7 (2.8)	14 (3.4)	8 (6.6)	0.177^c^
Marfan syndrome, n (%)	11 (4.4)	11 (2.7)	17 (14.0)	**< 0.001**^c^
Previous cardiac surgery, n (%)	7 (2.8)	10 (2.5)	4 (3.3)	0.870^c^
Coronary artery heart disease, n (%)	2 (0.8)	2 (0.5)	1 (0.8)	0.854^d^
Prior Stroke, n (%)	9 (3.6)	23 (5.6)	6 (5.0)	0.512^c^
Prior Shock, n (%)	1 (0.4)	11 (2.7)	1 (0.8)	0.062^d^
Prior Resuscitation, n (%)	1 (0.4)	6 (1.5)	2 (1.7)	0.398^d^
Extension of AAAD, n (%)				0.163^c^
Ascending aorta	12 (4.8)	13 (3.2)	3 (2.5)	
Aortic arch	99 (39.9)	137 (33.6)	38 (31.4)	
Descending aorta	9 (3.6)	20 (4.9)	2 (1.7)	
Abdominal aorta and/or beyond	128 (51.6)	238 (58.3)	78 (64.5)	
Time from symptom onset to surgery(h), median (IQR)	47.6 (39.6–55.6)	39.5 (33.7–45.4)	41.4 (29.2–53.7)	0.276^b^

^a^Analysis of Variance; ^b^Kruskal-Wallis tests; ^c^Chi-square test; ^d^Fisher’s exact test; *BMI* body mass index, *AAAD*, acute type A aortic dissection, *SD* standard deviation, *IQR* interquartile range

Patient preoperative, and operative variables are summarized in Table 2. The mean of systolic blood pressure (BP) was much higher in the obese group compared with the overweight and normal weight groups(147.1 ± 27.9 vs 143.6 ± 31.0 vs 133.7 ± 27.4, respectively; *P*<0.001), the same with diastolic BP (78.1 ± 15.7 vs 76.8 ± 17.8 vs 71.9 ± 14.7, respectively; *P*<0.001), and pulse pressure (69.0 ± 22.8 vs 67.0 ± 23.5 vs 61.7 ± 21.1, respectively, *P* = 0.003). The obese patients had higher white blood cell counts (WBC), hemoglobin, and serum creatinine compared to the normal weight and overweight patients (*P*<0.05). No significant differences were observed among other preoperative and operative variables (*P*>0.05).

**Table 2:** Preoperative, and operative data of stratified by BMI categories

Variables	normal weight group 18.5–23.9 kg/m^2^ (*N* = 248)	overweight group 24–27.9 kg/m^2^ (*N* = 408)	obese group ≥28 kg/m^2^ (*N* = 121)	*P*-value
**Preoperative**				
Systolic BP (mm/Hg), mean (SD)	133.7 ± 27.4	143.6 ± 31.0	147.1 ± 27.9	**< 0.001**^**a**^
Diastolic BP (mm/Hg), mean (SD)	71.9 ± 14.7	76.8 ± 17.8	78.1 ± 15.7	**< 0.001**^**a**^
PP (mm/Hg), mean (SD)	61.7 ± 21.1	67.0 ± 23.5	69.0 ± 22.8	**0.003**^**a**^
Heart rate, mean (SD)	80.7 ± 16.0	81.7 ± 17.3	83.6 ± 14.8	0.296^**a**^
WBC (×10^9^/l), mean (SD)	11.3 ± 3.8	12.9 ± 4.2	13.4 ± 4.4	**< 0.001**^**a**^
Neutrophil (× 10^9^/L), mean (IQR)	22.7 (19.0–26.4)	26.9 (23.8–30.0)	21.0 (16.4–25.6)	0.082^b^
Lymphocyte (× 10^9^/L), median (IQR)	2.6 (2.0–3.1)	2.6 (2.2–3.0)	2.3 (1.4–3.1)	0.734^b^
Platelet (× 10^9^/L), median (IQR)	184.3 (174.7–194.0)	182.1 (175.4–188.9)	189.4 (178.6–200.2)	0.607^b^
Creatinine (umol/L), median (IQR)	98.5 (92.0,105.0)	119.4 (108.7130.2)	99.8 (90.2109.4)	**0.005**^b^
Hemoglobin(g/l), mean (SD)	122.9 ± 18.5	128.5 ± 19.4	136.1 ± 18.0	**< 0.001**^**a**^
Serum albumin (g/dL), mean (SD)	3.5 ± 0.4	3.7 ± 0.6	3.8 ± 0.6	**< 0.001**^**a**^
BUN (mmol/L), median (IQR)	7.0 (6.5–7.4)	8.5 (6.1–10.9)	7.1 (6.6–7.7)	0.506^b^
**Operative**				
Operating time (min), median (IQR)	320.4 (309.2–331.7)	318.5 (309.8–327.2)	334.2 (315.7–352.7)	0.251^b^
CPB time (min), median (IQR)	168.9 (159.9–177.9)	162.8 (156.8–168.9)	172.9 (157.8–188.1)	0.288^b^
Aortic cross-clamping time (min), median (IQR)	80.6 (73.5–87.8)	80.7 (75.7–85.7)	72.4 (64.7–80.0)	0.265^b^

^a^Analysis of Variance; ^b^Kruskal-Wallis tests; *BMI* body mass index, *BP* blood pressure, *PP* pulse pressure, *WBC* white blood cell, *BUN* blood urea nitrogen, *CPB* cardiopulmonary bypass, *SD* standard deviation, *IQR* interquartile range

As shown in Table 3, the in-hospital mortality was no significant difference among the three groups(16.9% vs 21.8% vs 24.0%, respectively; *P* = 0.198), but there was a significant difference among three groups in MAO (62.9% vs 72.1% vs 77.7%, respectively; *P*<0.006). The duration of ICU stays was the longest in the obese group followed by the overweight and normal weight groups [12.0 (10.0, 14.1) vs 9.5 (8.6, 10.5) vs 8.1 (7.1, 9.2), respectively; *P* = 0.001], which was corresponded to the hospital stays [27.4 (24.3, 30.5) vs 22.6 (21.3, 23.9) vs 21.0 (19.8, 22.2), respectively; *P*<0.001]. No significant difference was present in stroke, myocardial infarction, low cardiac output syndrome, arrhythmia, aortic dissection rupture, respiratory insufficiency, acute renal failure, gastrointestinal bleeding, deep sternal wound infection, septicemia among the three groups(*P*>0.05) except prolonged MV (44.8% vs 55.6% vs 66.1%, respectively; *P*<0.001), and postoperative bleeding (0.8% vs 0.5% vs 3.3%, respectively, *P* = 0.024).

**Table 3:** Postoperative data of stratified by BMI categories

Variables	normal group 18.5–23.9 kg/m^2^ (*N* = 248)	overweight group 24–27.9 kg/m^2^ (*N* = 408)	obesity group ≥28 kg/m^2^ (*N* = 121)	*P*-value
**Postoperative**				
Duration of ICU stays (d), median (IQR)	8.1 (7.1,9.2)	9.5 (8.6,10.5)	12.0 (10.0,14.1)	**0.001**^a^
Duration of hospital stays (d), median (IQR)	21.0 (19.8,22.2)	22.6 (21.3,23.9)	27.4 (24.3,30.5)	**< 0.001**^a^
In-hospital mortality, n (%)	42 (16.9)	89 (21.8)	29 (24.0)	0.198^**b**^
Stroke, n (%)	15 (6.0)	22 (5.4)	9 (7.4)	0.700^**b**^
Myocardial infarction, n (%)	7 (2.8)	7 (1.7)	6 (5.0)	0.135^b^
Low cardiac output syndrome, n (%)	2 (0.8)	3 (0.7)	2 (1.7)	0.632^c^
Arrhythmia, n (%)	3 (1.2)	14 (3.4)	3 (2.5)	0.219^c^
Aortic dissection rupture, n (%)	3 (1.2)	5 (1.2)	0	0.474^c^
Respiratory insufficiency, n (%)	3 (1.2)	6 (1.5)	0	0.412^c^
Acute renal failure, n (%)	46 (18.5)	105 (25.7)	30 (24.8)	0.098^**b**^
Gastrointestinal bleeding, n (%)	17 (6.9)	41 (10.0)	12 (9.9)	0.356^**b**^
Deep sternal wound infection, n (%)	3 (1.2)	4 (1.0)	4 (3.3)	0.155^c^
Septicemia, n (%)	1 (0.4)	0	1 (0.8)	0.249^c^
Postoperative bleeding, n (%)	2 (0.8)	2 (0.5)	4 (3.3)	0.024^c^
Prolonged MV (> 48 h), n (%)	111 (44.8)	227 (55.6)	80 (66.1)	**< 0.001**^**b**^
In-hospital major adverse outcomes, n (%)	156 (62.9)	294 (72.1)	94 (77.7)	**0.006**^**b**^

^a^Kruskal-Wallis tests, ^b^Chi-square test; ^c^Fisher’s exact test; *BMI* body mass index, *ICU* intensive care unit, *MV* mechanical ventilation, *IQR* interquartile range

The results of the multivariable logistic regression analysis are presented in Table 4. It demonstrated a higher risk of in-hospital MAO in the overweight (ORs:1.475, 95%CI:1.006–2.162), and obese patients (ORs: 2.147, 95%CI:1.219–3.782) with reference to the normal weight patients. Besides, WBC (ORs:1.111, 95%CI:1.058–1.167), age (ORs:1.030, 95%CI:1.014–1.047), cardiopulmonary bypass (CPB) time (ORs:1.005, 95%CI:1.002–1.008), and prior stroke (ORs:7.525, 95%CI:1.740–32.552) were associated with MAO(*P* < 0.05).

**Table 4:** Preoperative and intraoperative risk factors of in-hospital major adverse outcomes as determined by multivariate logistic regression analysis

Variables	ORs	95% CI	*P-*value
BMI			**0.020**
18.5–23.9	**Reference**		
24.0–27.9	1.475	(1.006,2.162)	**0.046**
>28.0	2.147	(1.219,3.782)	**0.008**
Marfan syndrome	0.708	(0.317,1.580)	0.399
Serum albumin	0.983	(0.950,1.018)	0.342
Systolic BP	1.003	(0.997,1.009)	0.267
Heart rate	1.008	(0.997,1.019)	0.158
WBC	1.111	(1.058,1.167)	**<0.001**
Creatinine	1.002	(0.999,1.004)	0.139
Male	0.953	(0.619,1.467)	0.826
Age	1.030	(1.014,1.047)	**<0.001**
CPB time	1.005	(1.002,1.008)	**0.002**
Time from symptom onset to surgery	0.999	(0.996,1.002)	0.394
Extension of AAAD			0.503
Ascending aorta	**Reference**		
Aortic arch	1.783	(0.753,4.224)	0.189
Descending aorta	1.541	(0.481,4.934)	0.466
Abdominal aorta and/or beyond	1.449	(0.622,3.374)	0.390
Prior stroke	7.525	(1.740,32.552)	**0.007**
Prior resuscitation	2.783	(0.605,12.808)	0.189
Hemoglobin	0.994	(0.984,1.004)	0.258

*Abbreviations*: *BMI* body mass index, *BP* blood pressure,*WBC* white blood cell, *CPB* cardiopulmonary bypass, *AAAD* acute type A aortic dissection, *ORs* Odds ratios, *CI* confidence intervals

## Discussion

Several previous studies have investigated the impact of BMI on the prognosis of cardiac disease [10, 11], but the conclusions remain in dispute. The present study is innovative to demonstrate the impact of BMI on MAO in Chinese AAAD patients undergoing surgery. A total of 777 AAAD patients were included in the study, which we found that the incidence of in-hospital mortality was 20.6%. We observed BMI was independently associated with MAO of patients with AAAD, and patients who were overweight and obese were significantly longer in the duration of ICU stays and hospital stays compared with the normal group. Besides, WBC, age, CPB time, and prior stroke were significantly and strongly related to MAO in patients following AAAD surgery, even after adjusting for other risk factors. Therefore, BMI was a useful marker to stratify the high-risk patients with AAAD.

It is unclear why overweight and obese affects the prognosis of AAAD patients, but variability in adherence to DNA methylation or adipose tissue in overweight and obese patients may explain our observations. Recently, Dr. Simone Wahl and colleagues carried out studies [17] about epigenome-wide association amongst 5387 individuals from three different population, demonstrating that alterations in DNA methylation are more likely to be the consequence of overweight or obesity, which methylation loci are related to lipid and lipoprotein metabolism, substrate transport, and inflammatory pathways. In addition, large amounts of bioactive mediators can be released from the adipose tissue, affecting blood pressure, inflammation, and other changes, and lead to endothelial dysfunction and arteriosclerosis [18]. Based on these analyses, it could be concluded that overweight and obese may affect postoperative inflammatory reactions in AAAD patients, thus lead to an increase in mortality.

Although there is a huge amount of literature in support of the concept of the ‘obesity paradox’, which indicates that a protective effect or no effect was observed in overweight or even obese on adverse outcomes [6]. However, a recent network meta-analysis suggested that the ‘obesity paradox’ disappeared in Asians, which is consistent with our results [8]. Lio et al. reported that obese patients were more likely to develop postoperative mortality and adverse outcomes compared to non-obese patients [10]. Kreibich et al. pointed out that obesity was not significantly associated with postoperative adverse outcomes in AAAD patients [11]. Our findings indicated that overweight and obese are at increased risk of MAO in AAAD patients, which wasn’t consistent with Kreibich’s studies. The inconsistency might be explained by the three follows: first, BMI showed great differences in different racial persons and especially between the Caucasian and the yellow race [19]; second, the BMI cutoffs of underweight, normal weight, overweight and obese in Chinese are different from those in other countries; third, there are differences between the studies at baseline, for example, patients with acute aortic dissection (AAD) in China had an earlier onset about 10 years than the International Registry of Acute Aortic Dissection [20]. Finally, the body fat content of Asian people is generally higher than white people of the same gender, age, and BMI even if their BMI is less than 25 kg/m^2^, they are more likely to develop type II diabetes and cardiovascular disease [21]. Therefore, it is not surprising that the results are quite different.

In this study, we found that the longer ICU stay and the higher proportion of prolonged MV in the overweight and obese group, whose finding was compatible with those of other studies [22, 23]. The reasons could be as follows: (1) The proportion of smokers is higher in overweight and obese groups when compared to the normal group respectively. The decreased pulmonary function in smokers may lead to the inconsistency of baseline lung function on admission in different BMI groups. In this study, the group with a higher proportion of smokers has a higher incidence of prolonged MV. (2) The adipose tissue creates a number of estrogens in overweight and obese patients that play an important role in pulmonary hypertension and remodeling [24]. It generally focuses on the changes in pulmonary and chest wall structures, which led to the increase of residual lung volume and chest wall impedance, a decrease of lung compliance and ventilation driving force, and abnormal ventilation-perfusion [25]. Furthermore, reduction or loss of lung volume due to the weight of adipose tissue can occur in obese or overweight patients during operation [26], leading to impaired lung function and delayed extubation. And prolonged time on mechanical ventilation may result in prolonged ICU stay and vice versa [27]. In summary, medical staff should establish reasonable ventilation strategies to shorten the length of mechanical ventilation and ICU stay.

An important finding of this study is that the MAO in AAAD patients was associated with age. It has been reported that age was an established risk factor for perioperative adverse outcomes in AAAD patients [28, 29]. Then we have a deep analysis of reasons. In the first instance, elderly patients are often poorly performed having asymptomatic clinical features in comparison with the young patients which usually led to a delay in diagnosis and treatment. It may also reflect that patients do not obtain timely medical attention [30]. Secondly, it is known that older patients often suffered from concomitant comorbidities, such as hypertension, coronary heart, diabetes, and a history of cardiac surgery. That means older patients are more serious when they arrive at the hospital, which may thereby lead to a worse surgical prognosis. Moreover, compared with younger, older patients were more seem to have a poor prognosis due to their low cardiopulmonary reserve, and the poor responses to cardiopulmonary bypass surgery, anesthesia, various drugs, and fluid infusion. The elderly people are more physically to have weakness [31, 32], which can cause physiological disorders including heart rate variability, systemic inflammatory states, a decline in immune function, and hormonal changes [33–35], and thus increased risk of mortality.

Another notable finding was that WBC, CPB time, and prior stroke were the independent factors for MAO. There are multiple lines of studies and clinical observations that suggest that the inflammatory process plays an important role in the progress and prognosis of aortic dissection [36]. WBC is a sensitive marker of the non-specific inflammatory response, demonstrated as a strong independent predictor of in-hospital clinical events for AAAD patients in previous studies [8, 37]. Elevated WBC count directly reflects acute inflammatory reaction and the extent of aortic injury in aortic dissection, which may result in an endothelial injury, procoagulant reaction, and microvascular injury, increase the release of vascular toxic factors and finally lead to an increased risk of adverse outcomes [38]. In addition, the CPB time is particularly associated with mortality in AAAD patients that has been observed by other investigators as well [39]. The longer the CPB time, the longer the organ is in the relative ischemic state, the greater the probability of ischemic injury after the operation, leading to serious complications and even death. We noted that patients who had a prior stroke had significantly higher adverse outcomes, as reported previously [40, 41]. This may be explained that these patients are necessary to guarantee sufficient cerebral perfusion due to a higher cerebral perfusion pressure during CPB, which is more vulnerable to hemodynamic instability. For the above reasons, the incidence of MAO will inevitably increase.

However, there are several limitations. First, this was a single-center retrospective study which has its inherent shortcomings, and the findings may not be generalizable to other regions. Second, the measure of body proportion relies solely on BMI which does not yield information on the distribution of body fat. Third, the follow-up period (30 days) in this study was relatively shorter, further studies with longer follow-up periods would be explored to provide additional insight into the impact of BMI on long-term prognosis. Finally, we excluded the underweight patients (<18.5 kg/m^2^) with a small sample size in order to avoid the error of the results, which may affect the results when their cases are large enough.

## Conclusions

In summary, the present study demonstrates that BMI is independently associated with higher MAO in patients who underwent AAAD surgery. And WBC, age, CPB time, and prior stroke are significant factors for MAO. The focus should be placed on overweight and obese patients in risk assessment before surgery. Further works need to provide additional insight into the impact of BMI on long-term prognosis.

## Data Availability

The datasets used and analyzed during the current study are available from the corresponding author on reasonable request.

## Abbreviations

MAO: Major adverse outcomes
BMI: Body mass index
AAAD: Acute type A aortic dissection
CPB: Cardiopulmonary bypass
SD: Standard deviation
IQR: Interquartile range
ANOVA: Analysis of variance
ORs: Odds ratios
CI: Confidence intervals
WHO: World Health Organization
SOFA: The Sequential Organ Failure Assessment
SPSS: The Statistical Package for the Social Science
AAD: Acute aortic dissection
WBC: White blood cell
BP: Blood pressure
PP: Pulse pressure
ICU: Intensive care unit
MV: Mechanical ventilation
BUN: Blood urea nitrogen
